# Detection of cervical high-grade squamous intraepithelial lesions and assessing diagnostic performance of colposcopy among women with oncogenic HPV

**DOI:** 10.1186/s12905-023-02538-2

**Published:** 2023-08-04

**Authors:** Xiaoxiao Li, Fenfen Xiang, Yunzhi Zhao, Qian Li, Qing Gu, Xinpei Zhang, Zixi Chen, Mengzhe Zhang, Jun Wang, Rongrong Liu, Xiangdong Kang, Rong Wu

**Affiliations:** 1 Laboratory Medicine Department, Putuo Hospital, Shanghai University of Traditional Chinese Medicine, Shanghai, China; 2 Department of Obstetrics and Gynecology, Putuo Hospital, Shanghai University of Traditional Chinese Medicine, Shanghai, China

**Keywords:** Colposcopy, HR-HPV, Cervical biopsy, High-grade squamous intraepithelial lesion

## Abstract

**Background:**

HPV screening tests may improve cervical cancer risk stratification and better guide decisions about follow-up with colposcopy/biopsy. This study aimed to estimate the risk of cervical intraepithelial neoplasia grade 2 or worse (CIN2+) among women with oncogenic HPV types and evaluate the performance of colposcopy in the diagnosis of histologic CIN2 + at Putuo Hospital, Shanghai, China.

**Methods:**

This cross-sectional survey was conducted from February 2020 to December 2022 among women who were referred to colposcopy. Women with high-risk (HR) HPV-positive, cytology testing and colposcopy-directed biopsy were included.

**Results:**

Univariate and multivariate analysis indicated that high-grade colposcopic impression ((OR, 17.61%, 95%CI: 11.54–26.85%) was associated with the highest risk for detecting CIN2+, followed by HSIL + cytology (OR, 6.90%, 95%CI: 3.56–13.37%) and HPV16/18 positive (OR, 2.91%, 95%CI: 2.12–3.99%). Overall, CIN2 + was detected in 14.6% of 2007 women. HPV16/18 had higher CIN2 + risks than other HR-HPV genotypes (30.1% vs. 10.2%, *P*<0.001). Among women with low-grade cytology, 24.1% had CIN2+, and the risks for HPV16/18 (58.2%) were higher than for other HR-HPV(16.8%). For those with high-grade cytology, there was no significant difference between HPV groups ( 75.0% vs. 72.9%, *P* > 0.05). The diagnostic performance of colposcopy in diagnosis of CIN2 + by senior and junior colposcopists was comparable.

**Conclusions:**

The results indicated that referral to colposcopy is recommended in managing women with HR-HPV positive, and colposcopic impressions provide key clues for identification CIN2+.

## Introduction

Cervical cancer remains an important public health for women, particularly in low-and middle-income countries, including China. Screening programs for cervical cancer, such as cytology and HPV testing, offer an opportunity to identify women who are at a higher risk of precancerous conditions [1, 2]. Studies have indicated that HPV screening is associated with a greater reduction in the overall incidence of cervical cancer compared to conventional cytology-based screening [3, 4]. Additionally, HPV testing demonstrates higher sensitivity than cervical cytology alone in detecting cervical intraepithelial neoplasia grade 2 (CIN2) or more severe cases (CIN2+) [5–7], leading to an increased number of positive HPV screening results and potential referrals for colposcopy referral [8]. CIN2 + might evolve to cervical cancer, and surgical treatment is the main treatment for CIN2+ [9]. The risk of recurrence after surgical treatment of CIN2 + is nearly 10% at 5 years, and positive endocervical margins and HR-HPV persistence are the main factors predicting the risk of recurrence [10, 11]. Therefore, early screening tests and colposcopy diagnosis are of great importance.

Colposcopy and biopsies are important diagnostic tools for workup for managing cervical cancer, allowing for the identification of size and location of precancer lesions. Several factors can influence the accuracy of colposcopy, such as the specific HPV genotype and cytology results, transformation zone (TZ) type, and the expertise of the colposcopist [12, 13]. It is worth noting that the performance of colposcopy is not fully standardized. Colposcopy-guided biopsy is often considered as the gold standard for diagnosing cervical precancers. Numerous studies have assessed the accuracy of diagnostic colposcopy in detecting high-grade squamous intraepithelial lesions [14–16]. To promote standardized colposcopic practice, the International Federation of Cervical Pathology and Colposcopy (IFCPC) proposed a more comprehensive terminology in 2011. The appropriate use of colposcopy can help reduce unnecessary invasive cervical biopsies. Therefore, it is important to evaluate the agreement and discrepancies between colposcopy findings and cervical biopsies in diagnosis high-grade lesions.

In our previous study, we found that among a large cohort of 23,866 women who underwent HPV screening at our laboratory, 11.65% were identified as having a high-risk of developing high-grade abnormalities [17]. However, there is currently a lack of data on the detection of histological abnormalities following colposcopy in women with high risk HPV genotypes. Therefore, the objective of this study was to evaluate the performance of colposcopy in diagnosing histological CIN2+, specifically focusing on the outcomes observed at Putuo Hospital. Additionally, we aimed to investigate the relationship between HPV genotype, cytology results and the presence of CIN2+.

## Materials and methods

### Study population

This is a retrospective cohort of women who underwent HPV testing for cervical screening and referred colposcopic examination from February 2020 and December 2022 at Putuo Hospital, a large tertiary center in Shanghai, China. Eligible for inclusion in this study were women older than 18 years of age and those who had been referred to a colposcopist because of abnormal screening results. Briefly, women who had complete HPV screening results, LCT reports and colposcopy-direct biopsy were included. To avoid the effect of reversing pathological results after treatment, we chose the first colposcopy results. For HPV genotypes, we chose the HPV test with the shortest interval from colposcopy. Women who had a hysterectomy or previous excisional treatment for CIN and those who underwent colposcopy but had no histologic diagnosis were excluded. Age, HPV screening result, cytology, transformation zone (TZ) types, colposcopic impressions, colposcopist’s level and histological results were retrieved from medical records. This study was conducted in accordance with the Declaration of Helsinki and was approved by the Institution Review Board of Putuo Hospital, Shanghai University of Traditional Chinese Medicine. As the retrospective analysis was based on anonymized data, the need for individual informed consent was waived.

### Liquid-based cytology and HPV testing

Cervical cell specimens were collected with cervical plastic brush and put into preservation solution ((Tellgen Life Science, Shanghai, China) for both liquid-based cytology (LCT) and HPV DNA tests. Cytology slides were interpreted by experienced cyto-technicians. Results were classified according to the Bethesda grading system (2014) [18], including no intraepithelial lesions or malignancy (NILM), atypical squamous cells of undetermined significance (ASC-US), low-grade squamous intraepithelial lesion (LSIL), atypical glandular cells of undetermined significance (AG-US), atypical squamous cells cannot exclude high-grade squamous intraepithelial lesion (ASC-H), high-grade squamous intraepithelial lesion (HSIL) or carcinoma. HPV DNA testing was performed on cobas^@^4800 platform (Roche Diagnostic, USA) [19] which detects HPV16, HPV18 and a pool of 12 other high-risk HPV genotypes (HPV31/33/35/39/45/51/52/56/58/59/66/68).

### Colposcopy and histological diagnosis

Colposcopic examinations were performed by different colposcopists based on the 2011 International Federation of Cervical Pathology and Colposcopy (IFCPC) [20]. Briefly, colposcopists with more than 10 years working experience were defined as senior colposcopists, and others were categorized as junior colposcopists. Histopathological outcomes were graded according to World Health Organization (WHO) terminology: normal, cervical intraepithelial neoplasia grade 1 (CIN1), cervical intraepithelial neoplasia grade 2 (CIN2), cervical intraepithelial neoplasia grade 3 (CIN3) and invasive carcinoma [3]. The accordance was the percentage of women diagnosed by colposcopy and histopathological findings.

### Statistical analysis

Data analysis were performed using Excel (version 2010) and SPSS software (version 22.0). Cervical precancer endpoint CIN2 + was defined for risk assessment. The immediate risk of CIN2 + was calculated by dividing the number of CIN2 + cases in all women in each subgroup and presented with binomial 95%CI. Risk was reported as a frequency percentage. Univariate and multivariate logistic analyses were employed to evaluate independent factors for the presence of CIN2 + with an enter approach. Odds ratios with 95% confidence interval (CI) were calculated. Accuracy, sensitivity, specificity, positive predictive value (PPV), and negative predictive value (NPV) were used to assess the diagnostic performance of colposcopy for CIN2+. We used a Chi-square test to examine the association between colposcopy and histologic findings. A *P-*value < 0.05 was considered statistically significant.

## Results

### Clinical characteristics of study population

**Fig. 1:**
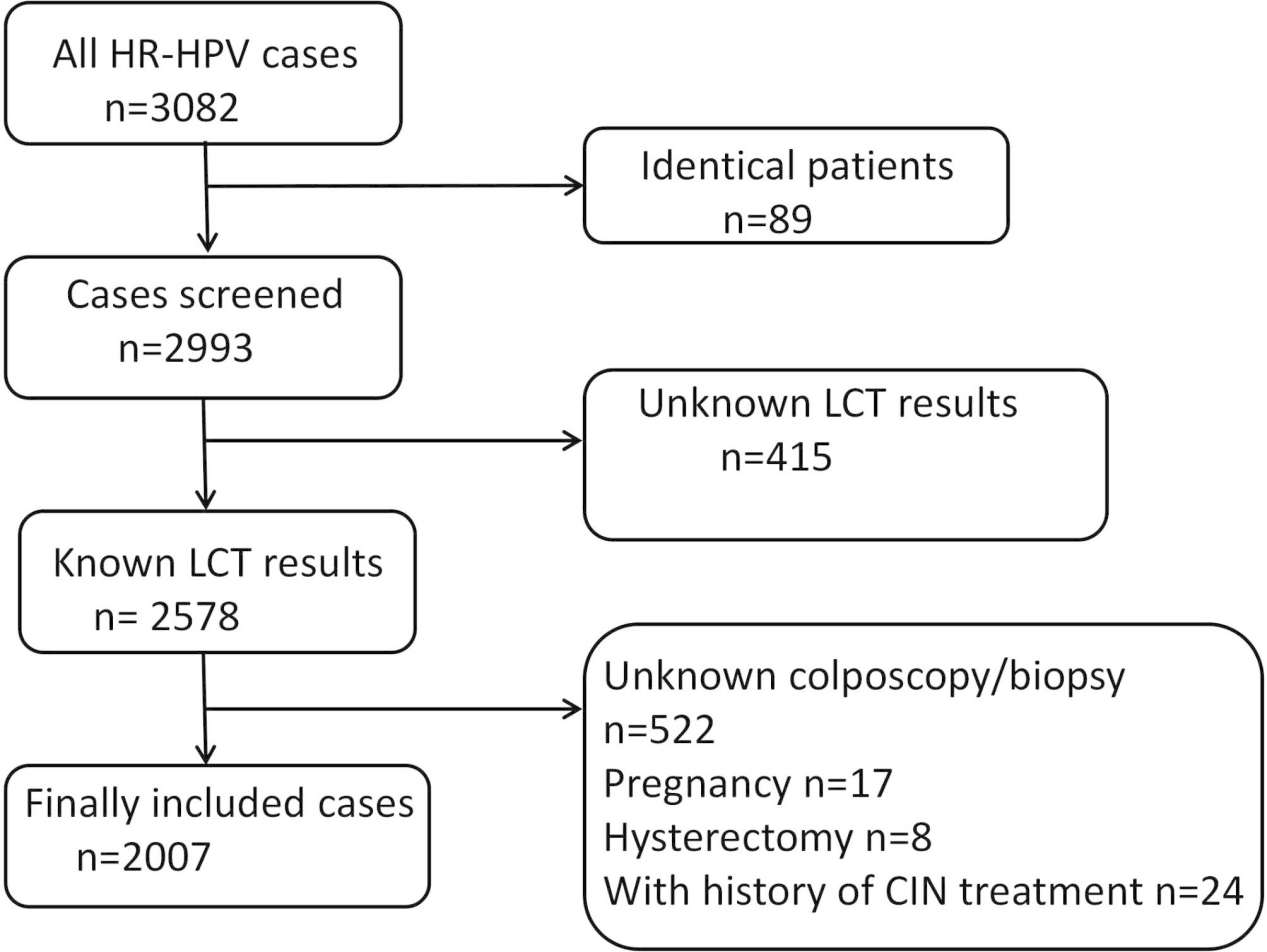
Flowchart illustrating the selection of study population

The selection of the study population is depicted in Fig. 1. Of 3082 women whose HPV tests were classified as high-risk during the study period. After excluding 89 duplicated patients, we further excluded patients who did not not meet the inclusion criteria. In total, 2007 women with oncogenic HPV screening results and referred to colposcopy were included in this study. The clinical information of colposcopy population was shown in Table 1. The mean age of the women was 42.4 ± 12.8 years (range 16 to 84 years), and the largest age group was aged ≤ 34 years. Among them, 473 (23.6%) were 16/18 positive, and 1534 (76.4%) were non-16/18 HR-HPV positive. For cytological reports, 1577 (78.6%) had normal cytology, 340 (16.9%) had low-grade cytology (LSIL, ASC-US and AG-US), and 69 (3.5%) had high-grade cytology (HSIL and ASC-H). Normal impression was the most common colposcopy diagnosis, followed by low-grade (32.6%) and high-grade colposcopy impression (12.8%). The final histological results included 1223 (60.9%) normal, 491 (24.5%) CIN1 and 293 CIN2+ (14.6%, 16 carcinoma).

**Table 1:** Summary of clinical characteristics among study women classified as high risk according to HPV genotyping

Characteristics	N	(%)
Total	2007	100
Age		
Mean ± SD	42.4 ± 12.8	
≤ 34	661	32.9
35–44	513	25.6
45–54	421	21.0
55–64	304	15.1
≥ 65	108	5.4
HPV status		
HPV16/18	473	23.6
Non-16/18 HR-HPV	1534	76.4
Colposcopy diagnosis		
Normal impression	1095	54.6
Low grade impression	656	32.6
High grade impression	256	12.8
Histology results		
Normal	1223	60.9
CIN1	491	24.5
CIN2/3	277	13.8
Carcinoma	16	0.8
Transformation zone		
TZ1	813	40.5
TZ2	578	28.8
TZ3	616	30.7
Cytology		
NILM	1577	78.6
LSIL/ASCUS/AGUS	340	16.9
HSIL+/ASC-H	69	3.5
Unsatisfactory	21	1.0
Colposcopist		
Junior	1372	68.4
Senoir	635	31.6

CIN: cervical intraepithelial neoplasia; TZ: transformation zone; NILM: negative for intraepithelial lesion or malignancy; LSIL: low-grade squamous intraepithelial lesion; ASCUS: atypical squamous cells of undetermined significance; AGUS: atypical glandular cells of undetermined significance; ASC-H: atypical squamous cells-cannot exclude HSIL; HSIL: high-grade squamous intraepithelial lesion

### Association between clinical factors and CIN2 + histological diagnosis

The associations between the above clinical factors and the histological diagnosis of CIN2 + by using univariate and multivariate logistic regression analysis were presented in Table 2. HPV16/18, HSIL + cytology and high-grade colposcopy impression were significantly correlated with an increased risk of detecting CIN2+. Women with high-grade colposcopy impression were at higher odds of detecting CIN2+ (OR, 17.61%, 95%CI: 11.54–26.85%) than HSIL + cytology (OR, 6.90%, 95%CI: 3.56–13.37%), low-grade colposcopy impression ((OR, 3.01%, 95%CI: 2.05–4.42%) and HPV16/18 positive (OR, 2.91%, 95%CI: 2.12–3.99%).

**Table 2:** Univariate and multivariate analysis of factors and their association with CIN2 + in the final histopathologic findings

Risk factors	Univariate analysis		Multivariate analysis	
	OR (95%CI)	*P* value	OR (95%CI)	*P* value
Age				
≤ 34	1.00		1.00	
35–44	1.31 (0.94–1.82)	0.111	1.72 (1.17–2.53)	0.006
45–54	1.14 (0.80–1.63)	0.466	1.55 (1.00-2.41)	0.049
55–64	1.22 (0.83–1.81)	0.306	1.80 (1.03–3.14)	0.039
≥ 65	1.66 (0.98–2.81)	0.061	2.63 (1.28–5.40)	0.008
HPV status				
Non-16/18 HR-HPV	1.00		1.00	
HPV 16/18	3.66 (2.83–4.75)	<0.001	2.91 (2.12–3.99)	<0.001
Cytology				
NILM	1.00		1.00	
LSIL	2.83 (2.10–3.82)	<0.001	2.13 (1.50–3.03)	<0.001
HSIL+	25.27 (14.41–44.32)	<0.001	6.90 (3.56–13.37)	<0.001
Colposcopy				
Normal	1.00		1.00	
Low-grade	3.66 (2.55–5.24)	<0.001	3.01 (2.05–4.42)	<0.001
High-grade	29.25 (20.03–42.73)	<0.001	17.61 (11.54–26.85)	<0.001
Transformation zone				
TZ1/2	1.00		1.00	
TZ3	0.55 (0.41–0.74)	<0.001	0.73 (0.46–1.16)	0.182
Colposcopist’s skills				
Junior	1.00		1.00	
Senior	1.12 (0.86–1.46)	0.392	1.23 (0.89–1.70)	0.206

CIN2+: cervical intraepithelial neoplasia grade 2 or worse; NILM: negative for intraepithelial lesion or malignancy; LSIL: low-grade squamous intraepithelial lesion (included atypical squamous cells of undetermined significance and atypical glandular cells of undetermined significance); HSIL+: high-grade squamous intraepithelial lesion or worse (included atypical squamous cells-cannot exclude HSIL). TZ: transformation zone

### Risks among oncogenic HPV-positive women referred immediately to colposcopy

The CIN2 + risk for women with low-grade cytology regardless of HPV genotype was 24.1% (95%CI: 19.6–28.7%). CIN2 + risks combination of HPV and cytology categories were shown in Table 3. Women with HPV16/18 positive low-grade cytology (58.2%, 95%CI: 46.4–70.0%) had a higher CIN2 + risk than women with other HR-HPV positive low grade cytology (16.8%, 95%CI: 12.4–21.3%) (*P*<0.001). However, the CIN2 + risk among women with HPV16/18 positive and high-grade cytology was 75.0% (95%CI: 60.0–90.0%), with no differences when compared to other ongenic HPV positive and high-grade cytology women (72.9%, 95%CI: 58.7–87.3%, *P* > 0.05).

**Table 3:** Distribution of high-grade squamous or worse lesions (CIN2+) detection according to HPV and LCT results

HPV result	LCT	N	CIN2+,N	Risk (95%CI)
Overall	NILM	1577	159 (10.1%)	8.6–11.6%
	LSIL/ASCUS/AGUS	340	82 (24.1%)	19.6–28.7%
	HSIL+/ASC-H	69	51 (73.9%)	63.6–84.3%
	Unsatisfactory	21	1(5.0%)	
HPV16/18	NILM	370	78 (21.1%)	18.5–27.4%
	LSIL/ASCUS/AGUS	67	39 (58.2%)	46.4–70.0%
	HSIL+/ASC-H	32	24 (75.0%)	60.0–90.0%
Non-16/18 HR-HPV	NILM	1209	82 (6.8%)	5.4–8.2%
	LSIL/ASCUS/AGUS	273	46 (16.8%)	12.4–21.3%
	HSIL+/ASC-H	37	27 (72.9%)	58.7–87.3%

NILM: negative for intraepithelial lesion or malignancy; LSIL: low-grade squamous intraepithelial lesion; ASCUS: atypical squamous cells of undetermined significance; AGUS: atypical glandular cells of undetermined significance; ASC-H: atypical squamous cells-cannot exclude HSIL; HSIL: high-grade squamous intraepithelial lesion

### Diagnostic performance of colposcopy for identifying CIN2+

The overall concordance rate was 60.0% (1205/2007). 365 cases were under-diagnosed. Specifically, 220 (60.3%) and 145 (39.7%) were finally diagnosed by histopathologic analysis with CIN1 and CIN2+, respectively. Considering colposcopy-directed biopsy as the gold standard, we evaluated the performance of colposcopy in the diagnosis of CIN2+. The overall accuracy, sensitivity, specificity, PPV, and NPV for diagnostic detecting CIN2 + were 87.4%, 50.5%, 93.7%, 57.8% and 91.7% (Table 4). There were no statistical differences for women diagnosed by junior and senior colposcopists except for PPV as shown in Table 4 (52.9% versus 71.0%, *P*<0.001).

**Table 4:** Performance of colposcopy in the detection of histologic CIN2+

Group	Accuracy (95%CI)	Sensitivity (95%CI)	Specificity (95%CI)	PPV (95%CI)	NPV (95%CI)
Overall	87.4% (85.9–88.8%)	50.5% (44.8–56.2%)	93.7% (92.5–94.8%)	57.8% (51.8–63.9%)	91.7% (90.4–93.0%)
Junior	86.6% (84.8–88.5%)	51.0% (44.0-58.1%)	92.5% (91.0–94.0%)	52.9% (45.8–60.1%)	92.0% (90.4–93.5%)
Senior	89.0% (86.5–91.4%)	49.5% (39.6–59.3%)	96.3% (94.7–97.9%)	71.0% (60.3–81.7%)	91.2% (88.8–93.5%)

CIN2+: cervical intraepithelial neoplasia grade 2 or worse; PPV: positive predictive value; NPV: negative predictive value

## Discussion

The overall incidence of histologic CIN2 + among women with positive certain oncogenic HPV genotypes was 14.6%, 30.1% with HPV16/18 and 10.2% with non-16/18 HR-HPV. Women who tested with HPV16/18 types had a higher proportion of CIN2 + compared with women who tested positive for other HR-HPV. It was in line with previous studies conducted in Norway [4] and Australia [21]. Cervical cancer management is based on cervical cancer screening, which aims to detect precancerous cervical lesions and reduce the incidence and mortality of cervical cancer-related. Screening methods have evolved from the cervical cytology-based primary strategy to HPV genotyping-based strategy testing [22]. Several countries pay attention to more sensitive HPV tests as the primary screening test [23–25]. For instance, a large prospective clinical study of HPV-based primary screening in the United States, also found that women with positivity of HPV16/18 had a higher risk than other HR-HPV genotypes for the detection of CIN2+ [26]. Moreover, HPV16 viral load can be served as a relevant biomarker to identify women at high risk for cervical precancerous lesions [27].

Liquid-based cytology seems still interchangeable, and HR-HPV detection combined with cytology was recommended for cervical cancer screening [28]. The proportion of CIN2 + increased with the grade of cytology, from 10.1% among NILM women to 73.9% among women with HSIL+/ASC-H cytology. In consistent with results of previous studies [29, 30], our data demonstrated that women with cotesting results of HPV16/18 and HSIL+/ASC-H cytology had the highest risk of high-grade cervical lesions (75.0%). While high risk was also observed in women with other HR-HPV and HSIL+/ASC-H cytology (72.9%). Compared with women in the HPV16/18 and LSIL/ASCUS/AGUS group, women with non-16/18 HR-HPV and LSIL/ASCUS/AGUS cytological results had a much lower risk of CIN2+ (16.8%). The different risk profiles we observed in the HPV genotyping in combination with cytology could increase the predictive value of HPV-based management and offer valuable information for risk stratification. As both HR-HPV testing and cytology have considerable false negative rates when used alone in the prediction of CIN2+, risk-based management algorithm by HR-HPV genotyping and cytology remains the most effective screening strategy for high-grade cervical lesions.

Colposcopy is a subjective examination method, and many studies have reported the accuracy of colposcopy in detecting cervical precancers and cancers in different groups of patients and various clinical settings [31–33]. It has been reported that colposcopic diagnosis often underestimates [31] or overestimates [34] in predicting squamous intraepithelial lesions. Recently, a systematic review and meta-analysis showed that the overall accuracy was 89% when using colposcopy to detect CIN2+, with combined sensitivity and specificity 68% and 93%, respectively [35]. Interestingly, they also demonstrated that colposcopy was more sensitive to low-grade squamous intraepithelial lesions or worse (LSIL+) and was more specific to CIN2+ [35]. The sensitivity of colposcopic diagnosis ranged from 29 to 100% and the specificity from 12 to 88% based on 11 studies [36]. In our study, we showed a comparable accuracy of colposcopy diagnosis (87.4%), with relatively lower sensitivity (50.5%) and raised specificity (93.7%) when CIN2 + was the threshold. Bai et al. [37] found that colposcopy had 69.7% accuracy in identifying HSIL + cases. In a similar study done in Bangladesh, the sensitivity and specificity of colposcopy correctly diagnosing histologic CIN2 + were 50% and 94% [38]. It should raise our great attention that nearly half of CIN2 + cases are missed at initial colposcopy based on our results. Previous studies have indicated that more than 30% cases of CIN2 + would progress into cervical cancers within a period of 10–15 years [39].

The performance of colposcopy in detecting cervical pathology may vary substantially between different experiences of colposcopists. Wei et al. [12] found that the accuracy and sensitivity of senior colposcopists were significantly higher than those of junior colposcopists. Conversely, Stuebs et al. [40] considered that there was no significant difference between colposcopists based on their experience. In our study, the performance of colposcopic accuracy by senior and junior colposcopists in detecting CIN2 + was comparable, which was in accordance with Dorji et al. findings [13]. We also noticed that our colposcopists had a PPV for histopathologic CIN2 + of 57.8%, lower than 69.9% reported by Chin et al. [21]. It is believed that the accuracy of the colposcopic diagnosis can be improved by strictly following the standardized colposcopy steps and regularly providing update courses and practices for colposcopists. On the other hand, it has been demonstrated that addition of colposcopic impressions can refine the management of women with abnormal screening results to avoid missing HSIL/CIN3 lesions [41].

For management of women with CIN2+, the current clinical guideline recommends immediate ablation or excisional surgical treatment. It is undeniable that surgical approach plays an important role in the treatment of CIN and cervical cancer. Nonetheless, previous studies also have indicated that excisional surgical methods in young women would produce adverse effects on pregnancy and neonatal outcomes [42, 43]. A systematic review has shown that surgical treatment (especially in Cold-Knife Conization and Large Loop Excision of Transformation Zone) of CIN was associated with an increased risk of preterm delivery, low birth weight and preterm premature rupture of membrane before 37 pregnancy weeks compared to untreated women [44]. Pecorino et al. [45] have reported that total laparoscopic radical hysterectomy had significantly longer operative time but lower intra-operative estimated blood loss compared to abdominal radical hysterectomy for cervical cancer. Several retrospective studies highlighted that minimally invasive laparoscopic radical hysterectomy correlated with improved short-term outcomes in comparison to open abdominal radical hysterectomy [46, 47]. However, these two approaches had similar survival and morbidity outcomes for early-stage cervical cancer according to long-term (ten years) follow-up [48].

A major strength of this study was the use of real-world data from a number of women who were HPV-positive and underwent colposcopy and biopsy examinations. The colposcopy was performed by clinicians and laboratory testing was analysed at the same licensed department, limiting inter-laboratory variation. Our study contributed results from a highly unique group of women attending colposcopy which provided baseline data, and way forward for improvement. However, there are several limitations should be considered. First, HPV-negative and without biopsy results women were excluded. This elimination may have introduced unknown bias in the analysis. Second, we presented the immediate risk of CIN2 + at a time point. A longer follow-up on those screen-positive patients and data from the second round of screening among negative women could reflect more accurate clinical practice and increase the precision of risk assessments. Third, the sample size for some categories was small and larger numbers are needed to make the results more robust. Finally, we have only studied colposcopic accuracy for detecting CIN2+, the data required to discern differences between CIN2+, CIN3 + and cervical cancer are still needed.

## Conclusions

In conclusion, we found that HPV16/18 has a higher risk for CIN2 + than other HR-HPV among women referred to colposcopy. Combination of cytology can offer valuable information for risk stratification in managing HPV-positive women. The sensitivity and specificity of colposcopy were lower than similar studies in other countries, thus the performance of colposcopy needs to be improved in our clinic.

## Data Availability

The datasets used and/or analysed during the current study available from the corresponding author on reasonable request.
